# Leveraging human precision cut lung slices for the study of human parainfluenza virus 3 infection

**DOI:** 10.1186/s12931-025-03335-1

**Published:** 2025-11-14

**Authors:** Olga Danov, Philippe Vollmer Barbosa, Helena Obernolte, Maximilian Fuchs, Patrice Guillon, Larissa Dirr, Ibrahim El-Deeb, Meik Kunz, Leonie Hose, Gaël Martin, Fabian Röpken, Anna Zimmer, Lavinia Neubert, Danny Jonigk, Hans-Gerd Fieguth, Franziska Dahlmann, Sabine Wronski, Mark von Itzstein, Thomas Tschernig, Armin Braun, Katherina Sewald

**Affiliations:** 1 Fraunhofer Institute for Toxicology and Experimental Medicine, Member of the German Center for Lung Research (DZL), Biomedical Research in Endstage and Obstructive Lung Disease (BREATH), Fraunhofer Cluster of Excellence Immune-Mediated Diseases CIMD, Member of Fraunhofer International Consortium for Anti-Infective Research (iCAIR), Nicolai-Fuchs-Str. 1, Hannover, 30625 Germany; 2 Hannover Medical School, Hannover, Germany; 3 Institute for Biomedicine and Glycomics, Griffith University Gold Coast Campus, Southport, Australia; 4 Member of Fraunhofer International Consortium for Anti-Infective Research (iCAIR), Gold Coast, QLD Australia; 5 KRH Clinics Hannover, Hannover, Germany; 6 Anatomy and Cell Biology, Saarland University, Homburg/Saar, Germany; 7 Institute of Pathology, University Medical Center RWTH University of Aachen, Aachen, Germany

## Abstract

Human parainfluenza virus 3 is a highly abundant RNA virus that primarily affects young children, the elderly, and immunocompromised individuals, leading to severe lower respiratory infections and pneumonia. Despite an urgent need of treatment options for these high-risk patients, neither a vaccine nor specific antiviral are currently approved. Blocking viral entry by targeting the viral surface glycoprotein haemagglutinin-neuraminidase (HN) has shown promising results in vitro and, to some extent, in vivo. However, to further evaluate these antiviral approaches for potential human application, a detailed understanding of early hPIV-3 infection and drug treatment mechanisms in human lung tissue is needed. In this study, we established a model for early hPIV-3 infection in human precision-cut lung slices (PCLS). We demonstrate specific infection of small airway epithelial cells followed by a distinct antiviral and inflammatory response marked by expression and secretion of type I, II and III interferons, chemokines such as IP-10 and ITAC, and pro-inflammatory markers IL-6 and TNF-α, but only limited induction of cytokines associated with high clinical severity, such as IL-8. Prophylactic treatment with two viral entry HN-inhibitors significantly reduced hPIV-3 viral load and inflammatory response after infection in the human lung tissue slices, demonstrating the high usability of the PCLS infection model for pharmacological assessment of novel antiviral drugs.

**Supplementary Information:**

The online version contains supplementary material available at 10.1186/s12931-025-03335-1.

## Introduction

Human parainfluenza viruses (hPIV) are enveloped, negative single-stranded RNA viruses, belonging to the *Paramyxoviridae* family [1]. They are classified into four serotypes: hPIV-1, hPIV-2, hPIV-3, and hPIV-4, which show different seasonal occurrences [2]. hPIV can lead to both upper and lower respiratory tract infections, primarily affecting young children, immunocompromised adults, and the elderly [3]. Of these, hPIV-1, and, to a much smaller extent, hPIV-2 are largely responsible for upper respiratory tract infection (URTI) leading to croup, and hPIV-3 and, with less abundancy, hPIV-4 are more commonly found in severe lower respiratory tract infection (LRTI) resulting in bronchiolitis and pneumonia, with a higher risk for immunocompromised patients and very young infants [4, 5]. Severe hPIV-3 infection can lead to pneumonia in recipients of hematopoietic stem cell transplant, resulting in acute mortality rates of up to 50% and 75% mortality at 6 months [1]. hPIV-3-infected lung transplant patients show an increased risk for acute or chronic allograft rejection, and hPIV-3 is also estimated to be responsible for 2.6% of Chronic Obstructive Pulmonary Disease (COPD) exacerbations [6].

hPIV-3 infection typically starts at the pseudostratified mucociliary airway epithelium of the nose and then spreads to the large and small airways within few days [7]. The severity of the disease is linked to its location; while mild symptoms are associated with upper respiratory tract infections, bronchiolitis and pneumonia can develop when the infection reaches the small airways and lung parenchyma [7]. This is correlated to a distinct host immune response upon infection. Pro-inflammatory mediators such as interleukin (IL)−6, IL-8, macrophage inflammatory protein (MIP)−1α/β, monokine induced by gamma interferon (MIG), and interferon-gamma induced protein (IP)−10 are upregulated. Additionally, “regulated on activation, normal T cell expressed and secreted” (RANTES) is detectable in nasal swaps from infected pediatric patients [8]. IL-8 has been observed to be a marker for disease severity, being more expressed more prominently in patients with LRTI compared to URTI.

Effective antiviral therapies are urgently required to treat such severe disease in populations most at risk of acute LRTI, including immunocompromised patients. However, there is currently no approved antiviral agent with demonstrable efficacy available [9].

One approach to antiviral treatment involves blocking of viral entry and release by targeting the viral surface glycoprotein haemagglutinin-neuraminidase (HN), which is essential for entry receptor binding and budding of new virions from the cell. Two extensively studied hPIV inhibitors directed against the viral haemagglutinin-neuramidase (HN), BCX 2798 (4-azido-4-deoxy-Neu5iBu2en, 1 and 4-deoxy-4-phenyltriazole-Neu5iBu2en, 2) derivatives of the HN’s physiological receptor, sialic acid, both showed significant antiviral efficacy in cell-based assays [10–12]. 2 was designed to target a unique feature of the hPIV-3 HN protein; the 216-cavity formed by movement of the flexible 216-loop. 2 is approximately sixfold more potent in enzymatic assays and in the normal bronchial epithelial cell line than the reference compound 1. The occupation of the 216-cavity by 2 has been confirmed by X-ray crystallography [13]. Despite these early successes, including in vivo proof-of-concept data in mice – where it has been proven to efficiently block infection, as supported by extensive literature [10] – the question remained whether this efficacy can be fully replicated in the context of human lung tissue, given the potential differences in physiological and metabolic responses between species. The objective of this study was to investigate the effects of 1 and 2 on early hPIV-3 infection in fresh primary human lung tissue, which has already been shown to provide valuable information about viral infection for other pathogens [14].

## Results

### Parainfluenza virus infects primary human lung tissue ex vivo

**Fig. 1:**
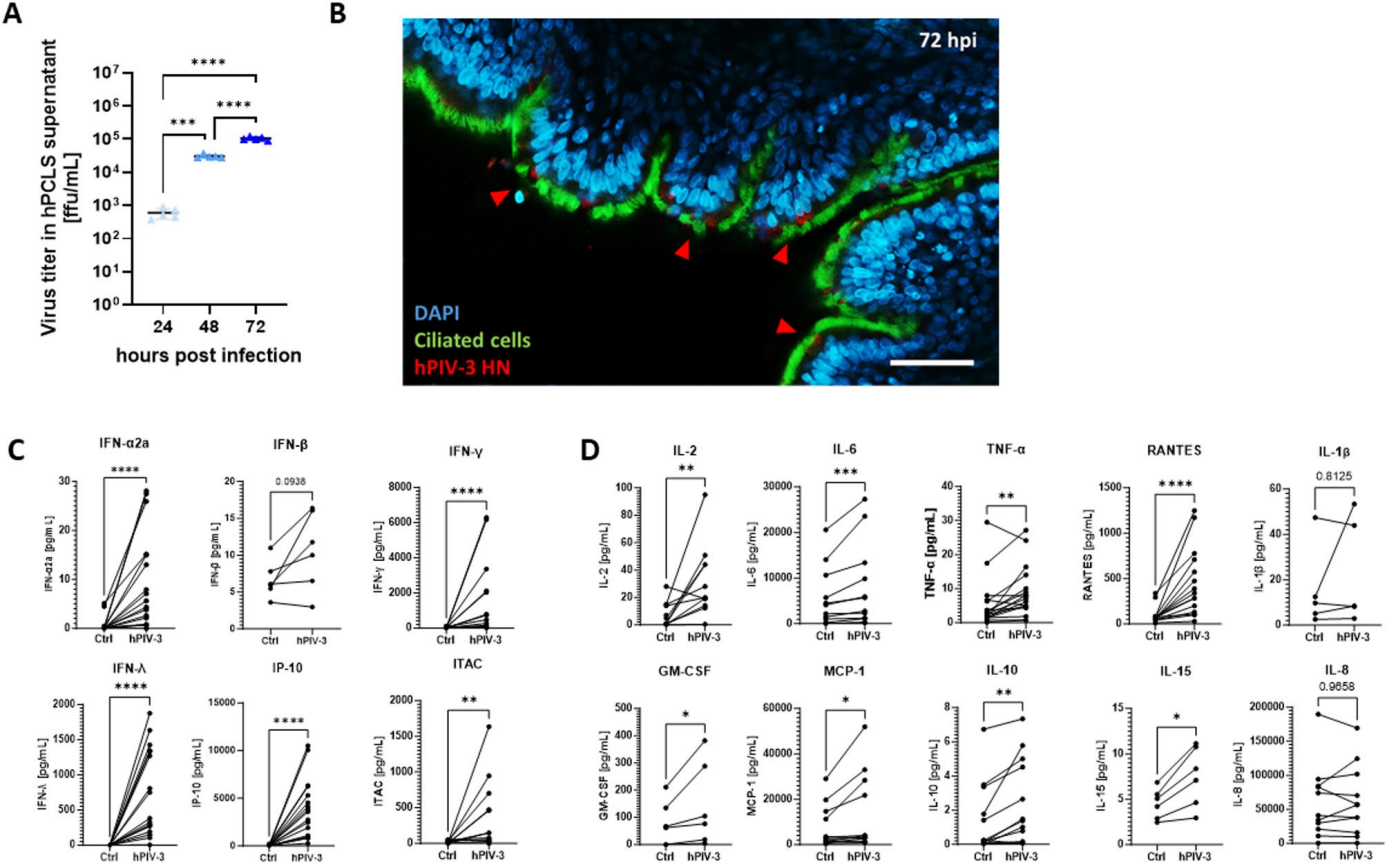
Parainfluenza virus infects and replicates in distal human lung tissue ex vivo inducing a specific antiviral and pro-inflammatory response. **A** hPIV-3 growth kinetics in human PCLS assessed by viral titer from PCLS supernatant at 24, 48, and 72 h.p.i., and measured by focus forming assay. 25,000 FFU were used for infection of human PCLS. *n* = 5 independent donors, *** *p* < 0.001, **** *p* < 0.0001 (One-way ANOVA with post-hoc Tukey´s multiple comparison test). **B** Confocal microscopic image of hPIV-3-infected human PCLS. PCLS were infected with hPIV-3 and fixed in PFA for successive staining at 72 h post infection. hPIV-3-HN, ciliated cell protein Arl13b, and nuclei (DAPI) were stained. Scale = 50 µm. Red arrows indicate infected patches of ciliated cells. **C** Induction of interferon response by hPIV-3 infection of human PCLS. Protein secretions of selected type I, II, III IFNs, and interferon-stimulated gene products were determined 72 h post infection. *n* = 17 for IFN α, γ, λ, and IP-10, *n* = 12 for ITAC, *n* = 6 for IFN β. Interferons and interferon-stimulated gene products were detected by multiplex kit from MSD or ELISA after hPIV-3 infection compared with uninfected PCLS (Ctrl). Cytokine release from lung tissue is displayed in pg/mL. Each data point represents one donor, *p < 0.05, **p < 0.005, ***p < 0.001, and ****p < 0.0001 (Wilcoxon matched-pairs signed rank test). **D** Induction of inflammatory response by hPIV-3 infection of PCLS. Protein secretion of selected inflammatory markers was determined 72 h post infection. *n* = 17 for TNF-α, and RANTES, *n* = 12 for IL-6, IL-2, MCP-1, IL-10, and IL-8, *n* = 6 for IL-1β, GM-CSF, IL-15. Inflammatory cytokines were detected by multiplex kit from MSD or ELISA after hPIV-3 infection compared with uninfected PCLS (Ctrl). Cytokine release from lung tissue is displayed in pg/mL. Each data point represents one donor, *p < 0.05, **p < 0.005, ***p < 0.001, and ****p < 0.0001 (Wilcoxon matched-pairs signed rank test)

Primary human lung tissue was infected with hPIV-3 strain C243. An increase in virus titer was observed over a time course of 72 h, confirming active replication of hPIV-3 in the human PCLS (Fig. 1A). Within the hPIV-3-infected PCLS, infected ciliated epithelial cells could be observed with partial co-localization of HPIV3-HN antigen and Cilia arl13B (Fig. 1B), indicating an active infection with hPIV-3 in these cells. Notably, the infection of airway epithelium occurred in patches rather than being homogeneously distributed (Supplementary Fig. 1 A). Interestingly, hPIV-3 did not induce a measurable cell lytic effect before 72 h post infection as shown by LDH release, which remained comparable between infected tissue and tissue inoculated with UV-inactivated hPIV-3 or medium until 96 h post infection, when hPIV-3 showed an increased LDH release (Supplementary Fig. 1B). This is in line with peaking viral load at 72 h post infection and no further increase after 96 h (Fig. 1A and Supplementary Fig. 1 A).

### Parainfluenza virus triggers a distinct pro-inflammatory and antiviral response in human lung tissue ex vivo

Infection of human lung tissue ex vivo with hPIV-3 resulted in a notable immunological shift. This was characterized by the release of both anti-viral and pro-inflammatory cytokines and chemokines, measured 72 h post-infection. The anti-viral interferon response was primarily marked by secretion of type I IFN-α (0.6 ± 1.5 vs. 9.4 ± 9.7 pg/mL) and type III IFN-λ (2.2 ± 2.5 vs. 730 ± 617 pg/mL)), while IFN-β did not reach statistical significance (Fig. 1C). Likewise, Type II IFN-γ (18.4 ± 30.8 vs. 1407 ± 2118 pg/mL), as well as interferon responsive chemoattractants IP-10 (37.32 ± 48.6 vs. 3856 ± 3080 pg/mL) and ITAC (18 ± 20 vs. 393 ± 496 pg/mL) were significantly secreted (Fig. 1C). Interestingly, peak protein of IFN-γ and IP-10 in supernatant could be observed after 72 h post infection (Supplementary Fig. 1 C). Analysis of pro-inflammatory cytokines showed a slight but significant increase of IL-6 (5400 ± 6486 vs. 7812 ± 9179 pg/mL), which also peaked at 72 h post infection (Supplementary Fig. 1 C), TNF-α (5.4 ± 7.4 vs. 8.9 ± 7.6 pg/mL), GM-CSF (79 ± 82 vs. 146 ± 154 pg/mL), and MCP-1 release (7445 ± 9255 vs. 12917 ± 16858 pg/mL) after hPIV-3 infection. T helper (Th) 1 response was further characterized by the significant induction of IL-2 (7.3 ± 8.8 vs. 31.6 ± 26.1 pg/mL) and RANTES (93.5 ± 92.4 vs. 453.8 ± 378.1 pg/mL) (Fig. 1D). While there was a slight increase in IL-15 secretion (5 ± 2 vs. 8 ± 3 pg/mL) and IL-10 (1.4 ± 2.0 vs. 2.3 ± 2.5 pg/mL) upon infection, IL-8 and IL-1β remained unchanged (Fig. 1D). Altogether, these results underscore the strong early immune response induced by hPIV-3 infection in tissue of the lower respiratory tract ex vivo. This response was shown to be replication-dependent, as UV-inactivated virus did not induce an enhanced immune reaction upon infection (Supplementary Fig. 1D). Furthermore, no secretion of asthma-associated cytokines such as IL-4 or IL-13 was observed in response to hPIV-3 infection (data not shown).

### Gene expression profiling in response to hPIV-3 infection: insights into immune responses and pathways in human lung tissue ex vivo

**Fig. 2:**
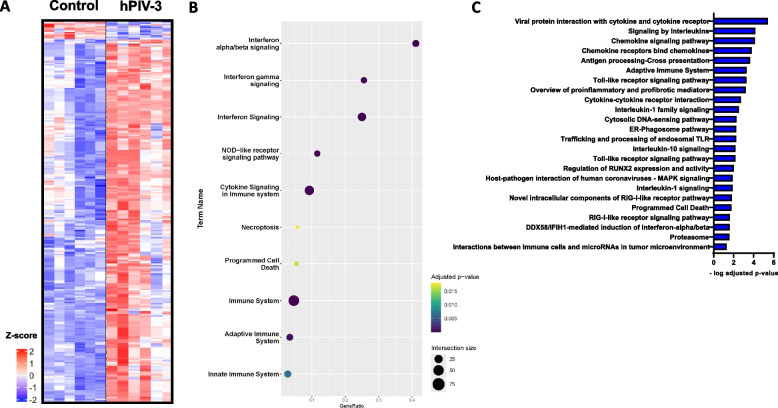
Bulk transcriptome analysis of hPIV-3-infected human precision-cut lung slices. **A** Heatmap displaying distinct clustering (red, upregulated; blue, downregulated) of hPIV-3-infected vs. uninfected control PCLS is displayed in heat map of differentially expressed genes. Within hPIV-3-infected human PCLS, 343 probes corresponding to 266 genes were upregulated, and 54 probes corresponding to 43 genes were downregulated, with a fold change greater than 1.5 and an uncorrected p-value of less than 0.05. **B** Pathway analysis of differentially expressed genes. Enrichment bubble plot shows overlap of Gene Ontology terms with differentially expressed genes of hPIV-3 infected human tissue samples. The analysis resulted in the identification of gene expression patterns associated with innate immune response and host defense mechanisms against viruses. **C** Detailed pathway analysis of upregulated genes induced by hPIV-3 infection in human PCLS. Functional over-representation analysis for pathways and Gene Ontology Terms was performed using the package “g:profiler2”

The analysis of whole genome transcriptomic data demonstrated a clear separation in gene expression profiles between in hPIV-3-infected PCLS and those treated with medium. This differentiation emphasized a consistent gene response to hPIV-3 across all donors, despite the expected donor-to-donor variability. Notably, hPIV-3-infection led to significant changes in the regulation of many genes related to innate and adaptive host immune response when compared with the medium treated samples (Fig. 2A). Among these genes, 343 probes corresponding to 266 genes were upregulated compared to medium treated controls, while 54 probes corresponding to 43 genes were downregulated, with a fold change greater than log 0.585 and an uncorrected p-value of less than 0.05.

The infection with hPIV-3 had a significant impact on large number of genes. Upon conducting pathway analysis using Gene Ontology terms, it became evident that the hPIV-3 virus induced gene expression associated with innate immune response and host defense mechanisms against viruses (Fig. 2B). Among the induced pathways, the most prominent were those related to the regulation of the immune system, and cytokine and interferon signaling which included both α/β and γ interferons. Another noteworthy finding was the strong up-regulation of the RIG-I-like receptor signaling pathway, which, although less prominent, showed high relevance in response to hPIV-3 virus infection in human PCLS (Fig. 2C). In addition to the antiviral immune response, genes responsible for the activation and regulation of immune cells were also detected, presumably mediated by infected epithelial cells. Pathways associated with the adaptive immune system, cytokine-cytokine receptor interactions, chemokine signaling pathways and the interleukin-1 family signaling were notably activated upon infection. This induction of key immune response pathways, observed 72 h after hPIV-3 infection, directly regulated processes like cytokine receptor binding, chemokine activity and the activation of cytokine genes such as *CCL2*, *RANTES*, *CXCL10/IP-10*, and *IL12B* (Fig. 2B).

In summary, the transcriptome data clearly point to the strong activation of a distinctive gene expression profile by hPIV-3 during the early stages of infection in primary human lung tissue, especially when compared to the UV-inactivated control, which also correlates to the observed cytokine and chemokine secretion pattern. Interestingly, hPIV-3 infection also triggered the expression of genes associated with necroptosis (*TNFSF10, STAT1, EIF2AK2, STAT2, TLR3, JAK2, CASP1, IRF9, MLKL*) and apoptosis (*TNFSF10, IRF1, PSMB9, PSMB8, CASP1, PSME2, PMAIP1, MLKL, PSMA4, PSME1, CASP4*) pathways. In contrast, only six genes were found to be downregulated upon infection, including *FAM101B*, *GXYLT2*, *TNFRSF10D*, *PPARGC1A*, *COL3A1*, and *KCNE4*. These have been shown to be involved in regeneration after viral infection and are also affected in other infections [15].

### Antiviral treatment with 4-phenyltriazole-Neu5iBu2en inhibits hPIV-3-induced infection and inflammation

**Fig. 3:**
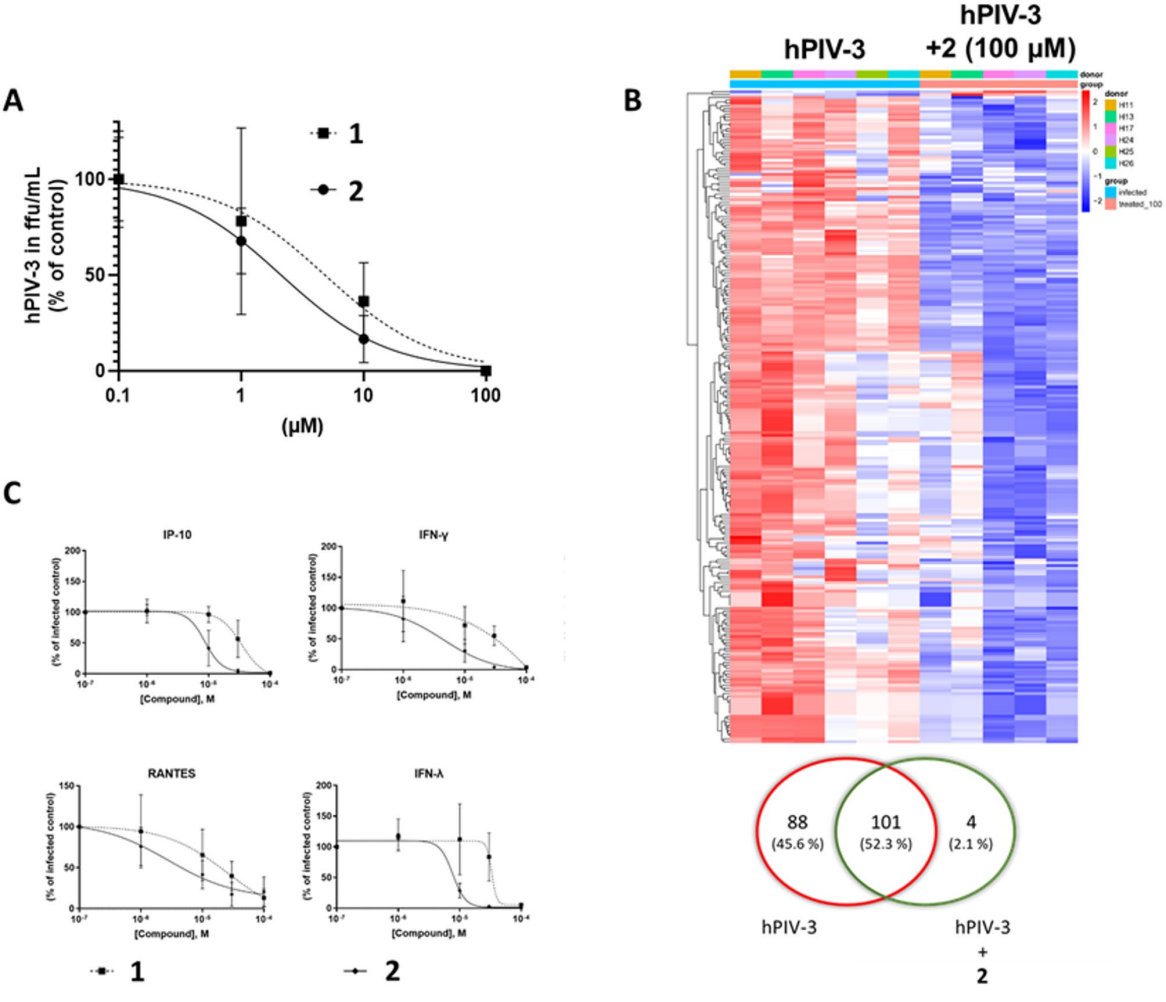
Viral inhibitors reduced viral replication in human PCLS ex vivo. Airway containing human PCLS were infected with hPIV-3 in presence of increasing concentrations of 1 and 2 or respective medium control. **A** Viral titer analyzed by focus forming assay and normalized to 100% control. Data are presented as mean ± SD for *n* = 4 independent donors (**2**) and *n* = 3 independent donors (1) with two technical duplicates each. **B** Transcriptomic analysis of differentially regulated genes showed prominent inhibition of hPIV-3-induced lung tissue responses ex vivo. Heat map of differentially regulated genes between treated (100 µM Inhibitor 2) and untreated hPIV-3 infected human PCLS. Venn diagram showing the distribution of differentially upregulated (> twofold change; *P* < 0.05) and downregulated genes (< twofold change; *P* < 0.05) by hPIV-3 infection. **C** Antiviral and pro-inflammatory cytokines were detected by multiplex kit from MSD or ELISA after hPIV-3 infection and dose-dependent treatment with 1 or 2 compared with hPIV-3 infected PCLS (Ctrl). Cytokine release from lung tissue is normalized to untreated infection control

To assess the efficacy of inhibiting viral replication, we conducted tests using two viral inhibitors 4-azido-Neu5iBu2en (1) and 4-phenyltriazole-Neu5iBu2en (2) in infected PCLS. 2 is an optimized derivative designed, in part, based on 1. As shown in Fig. 3A, treatment with both inhibitors resulted in a dose-dependent reduction in the release of progeny hPIV-3 viruses 72 h post-infection. The designer inhibitor 2 showed improved inhibition with an IC_50_ of 2.1 µM (*r*^2^ = 0.93, 95% CI [0.9–4.4 µM]), in contrast to 1, which had an IC_50_ of 4.3 µM (*r*^2^ = 0.92, 95% CI [1.2–14.7 µM]) in a prophylactic approach.

The dose-dependent reduction in virus load was accompanied by a decrease in cytokine release. Cytokine data confirmed the enhanced efficacy of 2 in inhibiting virus-induced cytokine release (Fig. 3C). High dose treatment led to a reversion of the cytokine release pattern after infection. This data suggests that no additional cytokine response was induced by high dose treatment alone.

To further evaluate whether reduced virus replication impacts gene expression profiles in human lung tissue, we analyzed transcriptomic profile in hPIV-3 infected PCLS treated with 10 µM and 100 µM 2. Treatment with the designer inhibitor resulted in a robust inhibition of the hPIV-3-induced tissue gene expression responses. At an inhibitor concentration of 100 µM, the number of hPIV-3-induced genes decreased from 189 to 105 genes with 101 genes overlapping (Fig. 3B). Remarkably, at an inhibitor concentration of 100 µM, no differentially expressed genes were found, suggesting the complete inhibition of hPIV-3 infection (data not shown). It further suggests that the compounds themselves have no effect on the expression levels of the investigated genes, as they do not induce any change compared to untreated. Canonical pathway analysis (workflow outlined in Supplementary Fig. 1E) showed that 2 prevented the initiation of the adaptive immune response and programmed cell death. However, it did not influence the induction of the necroptosis pathway at 72 h post infection.

## Discussion

Host defense responses elicited by hPIV-3 are critical in protecting the host against virus infection. Particularly, transplant patients, including solid organ, and hematopoietic stem cell transplantation recipients, often suffer from severe parainfluenza infections, which lead to a deadly outcome in 50% of cases [16]. Critically, whereas novel vaccination strategies show promising efficacy *in vivo* [17], these high-risk patients are often non-responsive towards vaccination and urgently need therapeutic options. However, the mechanisms responsible for activating the host response after hPIV-3 infection in humans are not fully understood. In this study, we investigated the effects of hPIV-3 infection on primary human lung tissue, which has been shown to provide valuable information on infection for other viruses by us and others [14, 18]. Our findings of hPIV-3 infection in human PCLS shed light on the host response to acute parainfluenza virus infection in the lower respiratory tract. We discovered that hPIV-3 infection induces an early robust host immune response and a specific antiviral gene expression profile in primary human lung tissue. We also profiled a designer antiviral inhibitor and demonstrated a successful reduction viral load upon treatment, resulting in a diminished immune response in infected tissue.

Epithelial cells serve as the first line of defense and are the primary host cells of hPIV-3 infection. Infected cells can initiate an antiviral state by continuously secreting antiviral factors to trigger defense responses against viral infections. Numerous studies using different respiratory viruses have shown that epithelial cells but also other cells such as macrophages can produce cellular restriction factors such as interferons in response to viral infection. We analyzed gene expression profiles of primary human lung tissue after 72 h of hPIV-3 infection. The results indicated that hPIV-3 infection differentially affected gene expression, primarily involving interferon, cytokine and chemokine signaling pathways in primary human lung tissue. To our knowledge, no transcriptome data from parainfluenza-infected tissue have been publicly available up to now. When comparing our results to other virus infections [19], we observed many similarities, demonstrating conserved antiviral responses in a virus-unspecific manner. We found that interferon-stimulated genes, crucial for restricting respiratory virus replication early after infection, such as CXCL10, CXCL9, CXCL11, OAS1, OASL, and ISG15, were significantly upregulated in PCLS 72 h post-infection. Notably, CXCL10, CXCL9, and CXCL11 had previously been found to be upregulated in human nasal epithelial cells after infection with human influenza H3N2 [20]. Our transcriptomic data revealed a more pronounced induction of type I interferons (IFN-α and IFN-β) and type II interferons (IFN-γ) compared to type III interferons (IFN-λ). However, on the protein level, the induction of IFN-γ and IFN-λ was more prominent than the slightly induced IFN-α and unchanged IFN-β release. The induction of type III interferon may represent a significant event within the lower respiratory tract tissue infected with parainfluenza, as it constitutes a beneficial antiviral response, as previously observed after influenza virus infection [21, 22].

hPIV-3 primarily affects younger children and ranks second, after respiratory syncytial virus (RSV), as a cause of bronchiolitis and pneumonia in children under 6 months [23]. In children, hPIV-3 infection has been reported to trigger inflammatory cytokines, such as IL-6, IL-8, MIP-1α, MIP-1β, MIG, RANTES, and IP-10 [8]. These data align with the cytokine response detected in PCLS, confirming the clinical relevance of lung tissue ex vivo. Interestingly, IL-8, which has been shown to be correlated to disease severity, was not significantly secreted upon infection, which might be attributed to the early stage and mildness of the PCLS infection model. This is in line with other markers for severity which were not upregulated in the model, e.g., OLAH [24].

Cell death plays an important role in the host's response to viral infections. Apoptosis and necroptosis are recognized as essential components of host defense mechanisms, eliminating pathogen-infected cells [25]. Parainfluenza virus infection in human lung tissue damages airway epithelium, as indicated by enriched apoptosis and necroptosis pathways contributing to cell death and barrier loss during infection. Necroptosis, a tightly regulated process induced by inflammatory stimuli or pathogens, has been shown to result in a highly inflammatory response [26]. Although these pathways were activated in our analysis, there was no corresponding increase in LDH release into the culture medium. This could be attributed to a relatively small proportion of respiratory airway epithelial cells infected with hPIV-3 in PCLS, insufficient to markedly elevate LDH levels, yet adequate to influence pathway analysis. Alternatively, it may reflect a delayed cellular integrity response to the initiation of necroptosis.

Despite hPIV-3's known role in exacerbating asthma and COPD in patients, our datasets did not reveal a gene overlap or cytokine induction that suggest an exacerbation phenotype. This is different to recent data for RV1b, which showed a significant overlap with genes associated with asthma and COPD in PCLS [27].

Nearly two decades ago, the initial efficacy of HN inhibitor 1 against hPIV-3 virus was demonstrated [11]. This encouraged activities in further antiviral compound development. The discovery of the viral HN protein's 216-loop [13] led to the design of neuraminic acid-based inhibitors, like 2, which exhibited improved efficacy and lower IC50 values compared to 1 in LLC-MK2, A549, and NHBE cells [12]. We evaluated 1 and 2 for antiviral treatment during acute hPIV-3 infection in human lung tissue. They dose-dependently reduced viral load, with 2 showing enhanced efficacy, confirming prior in vitro findings [12]. This reduced viral load positively affected the virus-induced inflammatory immune response. Interestingly, although the number of differentially expressed genes (DEGs) in mapped pathways decreased, pathways to necroptosis remained unaltered.

In general, primary human precision-cut lung slices (PCLS) offer highly relevant data about immune responses in the peripheral lung. However, like any model, it has limitations. Lung tissue lacks the blood and lymph systems, limiting immune cell influx. Still, it contains resident immune cells, a feature missing in primary epithelial cell cultures. However, only cells with the hPIV-3 entry receptor could be infected in the tissue slices, ensuring controlled infections in human PCLS.

In summary, hPIV-3 virus infection triggers a strong immune response in primary human lung tissue, mainly involving interferon and cytokine pathways. This response is vital in defending against the virus. However, the mechanism behind this response needs further investigation. Finally, we found that the promising antiviral inhibitor 2, significantly reduces viral load and the associated immune response in the human PCLS model. Our study further confirms the clinical relevance of this lung tissue model for researching viral infections.

### Funding

This work was supported by Biomedical Research in the Endstage and Obstructive Lung Disease Hannover (BREATH), Member of the German Center for Lung Research (DZL), Fraunhofer International Consortium for Anti-Infective Research (iCAIR), Bundesagentur für Sprunginnovationen (Sprin-D), and Fraunhofer Cluster for Immune-mediated Diseases (CIMD) Competence platform “Alternative methods to animal testing”. LD (NHMRC Early Career Fellowship, GNT1157150) and MvI (GNT1196520 and GNT2009677) gratefully acknowledge the National Health and Medical Research Council (NHMRC) for financial support.

## Methods

### Ethics statement

Human lung lobes were acquired from patients who underwent lobe resection for cancer at KRH Klinikum Siloah-Oststadt-Heidehaus (Hannover, Germany) or Hannover Medical School (MHH, Hannover, Germany). These experiments were approved by the ethics committee of the Hannover Medical School (MHH, Hannover, Germany) and in compliance with *The Code of Ethics of the World Medical Association* (number 2701–2015). All patients or their next of kin gave written informed consent for the use of explanted lung tissue for research.

### Media, chemicals and reagents

Dulbecco’s modified Eagle’s medium/nutrient mixture F-12 Ham (DMEM, pH 7.2–7.4) with L-glutamine and 15 mM HEPES without phenol red and fetal bovine serum was purchased from Gibco (Life technologies, Darmstadt, Germany). Medium for cultivation was supplemented with 100 units/mL penicillin and streptomycin, which were purchased from Lonza (Verviers, Belgium). Dulbecco´s phosphate-buffered solution without Ca^2+^ and Mg^2+^ (DPBS) was also purchased from Lonza (Verviers, Belgium). Low gelling temperature agarose, Avicel® PH-101, Earle’s Balanced Salt Solution (EBSS), protease inhibitor cocktail P1860, and Triton X-100 were supplied by Sigma-Aldrich (Munich, Germany). Antibodies used for immunofluoresence staining were mouse-anti-hPIV-3 HN (Fitzgerald Industries, Acton, USA)), rabbit polyclonal anti-arl13b (Thermo Scientific, Schwerte, Germany), donkey-anti-rabbit Cy2 (Jackson ImmunoResearch, Pennsylvania, USA), goat anti-Mouse IgG-HRP (Bio-Rad, Germany) and donkey-anti mouse Cy5 (Jackson ImmunoResearch, Pennsylvania, USA). Donkey serum was supplied by Dianova GmbH (Hamburg, Germany). DAPI was purchased from Invitrogen (Life technologies, Darmstadt, Germany). Paraformaldehyde was obtained from Sigma-Aldrich, Munich, Germany. TrueBlue™ substrate was obtained from Medac Diagnostika, Wedel, Germany. Inhibitors 1 and 2 were prepared in house using published methods (Reference Guillon et al. [21]).

### Cell culture

LLC-MK2 Derivative (ATCC® CCL-7.1™) were purchased from American Type Culture Collection (LGC Standards GmbH, Wesel, Germany) and maintained at 37 °C, 5% CO_2_ in DMEM supplemented with 1% penicillin/streptomycin, 2 mM glutamine (Gibco, 25030–024, Life Technologies, Darmstadt, Germany) and 10% heat-inactivated FBS (Sigma-Aldrich, F7524, Darmstadt, Germany).

### Preparation of human PCLS

Lung tissue was acquired from patients who underwent partial resection due to lung cancer at the Hannover Medical School (MHH, Hannover, Germany) or KRH Klinikum Siloah-Oststadt-Heidehaus (Hannover, Germany). Only tissue from macroscopically and microscopically tumor free parts of the lung were used for experiments. Human lung slices with peripheral airways were prepared as described before [28]. Briefly, a lung lobe was inflated with 2% agarose/medium solution. After the polymerization, the lung lobe was cut into slabs and PCLS of 8 mm in diameter were cut into 300 µm thin slices using the Krumdieck tissue slicer (Alabama Research and Development, Muniford, AL, USA). Tissue slices were cultivated submerged in medium (DMEM/F12 supplemented with 1% penicillin/streptomycin) at 37 °C, 5% CO_2_ overnight.

### Virus purification

LLC-MK2 cells (rhesus monkey kidney epithelial cells) at 90% confluency were inoculated with hPIV-3 strain C-243 at a multiplicity of infection of 0.01. Virus was amplified for 3 days at 35 °C, 5% CO_2_ in DMEM supplemented with 1% penicillin/streptomycin, 2 mM glutamine. Virus-containing culture supernatant was harvested, clarified at 3,000 × g for 15 min and loaded onto 30–60% non-linear sucrose gradient in GNTE buffer (200 mM glycine, 200 mM NaCl, 20 mM Tris–HCl, 2 mM EDTA, pH 7.4). After ultracentrifugation in Optima L80-XP Series ultracentrifuge equipped with a SW32 Ti rotor (Beckman Coulter, CA) at 4 °C, and 25,000 rpm for 2.5 h, the viral particles were collected between the 30–60% interface, resuspended in GNTE buffer, aliquoted and frozen at −80 °C.

### Viral titer

The viral titer was quantified by Focus Forming Assay (FFA) as previously described [12, 29]. Briefly, confluent LLC-MK2 cells in flat-bottom 48-well plate were washed and infected with 90 μL of serially-diluted virus (DMEM supplemented with 1% penicillin/streptomycin, 2 mM glutamine without FBS). Virus was allowed to adsorb to cells for 1 h at 35 °C, with gentle shaking every 15 min. Then, the inoculum was replaced with 300 μL of media containing 1% Avicel® for 36 to 40 h. The supernatant was removed, and cells were fixed with 300 μL of 2% paraformaldehyde at room temperature for 20 min and washed 3 times in PBS for 4 min. Then, 150 μL of 0.3% H_2_O_2_ and 0.5% Triton X-100 were added in each well and incubated at 37 °C under humidified atmosphere of 5% CO_2_ for 30 min. The washing was repeated, and the cells were stained with primary antibody (1:2,000) (mouse monoclonal IgG anti-hPIV-3-HN) diluted in 5% milk/PBS at 37 °C for 1 h. Subsequently the cells were washed with 0.02% Tween 20/PBS 3 times for 4 min, and 50 μL of the secondary antibody (1:3,000) diluted in 5% milk/PBS were added and incubated at 37 °C for 1 h. Cells were washed again and overlayed with 150 μL of TrueBlue™ substrate until dark blue foci appeared. The substrate was removed, and the plate was rinsed with tap water and air dried. The foci were manually counted and averaged between the triplicates and expressed as focus forming units (ffu) per mL.

### Infection of PCLS with hPIV-3 and antiviral treatment

PCLS were inoculated with 25,000 ffu/well in 250 µL of hPIV-3 (strain C243, ATCC VR-93). Infected PCLS were incubated at 35 °C and rocked every 15 min during inoculation to enable homogenous virus infection. Afterwards, the inoculum was discarded, and 500 µL of fresh medium were added to the PCLS. After incubation for 72 h, supernatant was collected for virus detection, LDH release, and cytokine quantification. Samples for the cytokine measurements were supplemented with 0.2% protease inhibitor cocktail (P1860, Sigma-Aldrich, Munich, Germany) and stored at −80 °C until analysis.

PCLS were treated with 25 µL of either 4-azido-4-deoxy-Neu5iBu2en (1) or 4-deoxy-4-phenyltriazole-Neu5iBu2en (2) in H_2_O and 5% DMSO resolved in a total volume of 250 µL medium (for a final concentration of 0.5% DMSO), or a respective medium control during inoculation by premixing 20 min prior to infection. Preincubated virus was used for inoculation of the PCLS for 1 h as described above. After withdrawal of the inoculum and single rinsing with PBS, PCLS were cultured in 500 µL medium for another 72 h.

### LDH assay

LDH release assay was performed according to the manufacturer’s instructions using Pierce™ LDH Cytotoxicity Assay Kit obtained from Roche (Mannheim, Germany). Thus, 50 µL of culture supernatant was incubated with 50 µL of reagent mix (diluted 1:46) in duplicates at RT in the dark for 20 min. The absorbance was measured at 492 nm using a microplate reader infinite F200Pro (Tecan, Männedorf, Switzerland). The reference absorption at 630 nm was subtracted from 492 nm.

### Cytokine measurement

Cytokines were measured using a multiplex panel (IFN-α2a, IFN-β, IFN-λ, TNF-α, IFN-γ, GM-CSF, IL-6, monocyte chemoattractant protein 1 (MCP-1), interferon-inducible T cell alpha chemoattractant (I-TAC), IL-15, IL-2, regulated on activation normal T cell expressed and secreted (RANTES), IL-10) (MSD, MesoScale Discovery, Gaithersburg, USA) assay. The MSD assay was performed following the manufacturer’s instructions using an MSD Sector Imager 2400. Cytokine concentrations were calculated using the Discovery Workbench software (version 4.0, Mesoscale Discovery, Gaithersburg, MD, USA) and based on a four-fold serial diluted standard. CXCL10/IP-10 was quantified using a commercially available ELISA (R&D Systems, Wiesbaden, Germany) according to the manufacturer’s instruction.

### Immunofluorescence staining and imaging

Paraformaldehyde-fixated PCLS were washed with PBS and permeabilized with 0.3% Triton X-100. PCLS were blocked with 4% donkey serum in PBS for 30 min and incubated with primary antibodies (mouse monoclonal IgG anti-hPIV-3-HN (1:100) and rabbit polyclonal anti-arl13b (1:100)) diluted at 4 °C in 4% donkey serum overnight. PCLS were washed three times and incubated with the secondary antibodies conjugated to Cy2 (1:100) and Cy5 (1:200) diluted in 4% donkey serum at room temperature for 2 h. DAPI was diluted 1:400 in PBS and PCLS were stained at room temperature for 45 min. Tissue slices were embedded with ibidi mounting medium and stored at 4 °C prior to imaging. Images were acquired using a confocal microscope LSM 800 (Carl Zeiss, Jena, Germany) with × 63 objectives. Z-stacks of 30 µm thickness were imaged with image resolution of 2048 × 2048.

### RNA isolation and transcriptome analysis

PCLS were flash frozen 72 h post infection and total RNA including miRNA was isolated as described before. RNA quantity was determined by spectrophotometry and quality was assessed using an Agilent 2100 Bioanalyzer (Agilent, South Plainfield, NJ) to determine the RNA integrity number (RIN). All samples had acceptable RIN numbers (range 4.9–10, average 7.4). Total RNA was converted into double-stranded cDNA, amplified and Clariom S (Affymetrix, Santa Clara, CA) were performed and analyzed as detailed in the online supplement. Data procession and analysis was performed within R version 4.1.3. Raw CEL files were loaded using the “oligo” package.

Data was normalized using the robust array average (RMA) method. Differential expression analysis was performed using linear models for microarray analysis (limma).

Genes with an adjusted *p*-value > 0.05 and log_2_FC >|0.585| were considered differentially expressed. Functional over-representation analysis for pathways and Gene Ontology Terms was performed using “g:profiler2” package.

### Statistical analysis

For statistical analysis the program GraphPad Prism (Version 9.3.1) was used. All treatment groups were compared with their respective controls.

Wilcoxon matched-pairs signed rank test was performed for comparison of two groups. For multiple comparisons of means, one-way ANOVA and post-hoc Tukey´s multiple comparison test was performed. Differences were considered to be statistically significant when *p* ≤ 0.05.

## Supplementary Information

Supplementary Material 1: Figure S1. A Exemplary confocal microscopy images of human PCLS infected hPIV-3 and UV-inactivated virus at 6, 24, 48, 72, and 96 hours post infection. hPIV-3-HN, ciliated cell protein Arl13b, and nucleiwere stained. Scale = 50 µm. Red arrows indicate infected patches of ciliated cells. B hPIV-3 infection only causes a cytopathic effect in human precision-cut lung slices after 96 hours post infection. LDH release from human tissue slices was measured 6, 24, 48, 72, and 96 hours post infection with medium control, UV-inactivated, or unmodified hPIV-3. An increased LDH release in infected slices compared to medium and UV control could be observed after 96 hours. C Kinetics of interferon response towards the infection.IFN-γ, IP-10, and IL-6 protein release from human tissue slices were measured 6, 24, 48, 72, and 96 hours post infection with medium control, UV-inactivated, or unmodified hPIV-3, via ELISA. Peak protein levels were observed after 72 hours post infection. b UV-inactivation renders hPIV-3 unable to induce an antiviral and pro-inflammatory response in human precision-cut lung slices. An aliquot of hPIV-3 was UV-inactivated during simultaneous cooling for 2 hours. The infection was performed according to non-inactivated virus. Cytokine secretion of selected cytokineswas measured from PCLS of 6 independent human donors. No significant increase in cytokine release was observed between UV-inactivated and non-infected control for any of the cytokines. E Exemplary ontology analysis outline. Raw data were processed and over-representation analysis for pathways and Gene Ontology Terms was performed using the package “g:profiler2”.

## Data Availability

No datasets were generated or analysed during the current study.

## References

[1] Branche AR, Falsey AR, Singh SK. Parainfluenza virus infection. 2016.10.1055/s-0036-1584798. 10.1055/s-0036-1584798 PMC7171724 27486735

[2] Glezen WP, Frank AL, Taber LH, Kasel JA. Parainfluenza virus type 3: seasonality and risk of infection and reinfection in young children. J Infect Dis. 1984;150:851–7. 6094674 10.1093/infdis/150.6.851

[3] Wu KW, et al. Clinical and epidemiological characteristics of human parainfluenza virus infections of children in southern Taiwan. J Microbiol Immunol Infect. 2018;51:749–55. 28757139 10.1016/j.jmii.2016.08.017

[4] Henrickson KJ. Parainfluenza viruses. Clin Microbiol Rev. 2003;16:242–64. American Society for Microbiology (ASM). 12692097 10.1128/CMR.16.2.242-264.2003 PMC153148

[5] Chellapuri A, et al. Human parainfluenza 2 & 4: clinical and genetic epidemiology in the UK, 2013–2017, reveals distinct disease features and co-circulating genomic subtypes. Influenza Other Respi Viruses. 2022;16:1122. 10.1111/irv.13012 PMC9530586 35672928

[6] Mohan A, et al. Prevalence of viral infection detected by PCR and RT-PCR in patients with acute exacerbation of COPD: a systematic review. Respirology. 2010;15:536–42. 20415983 10.1111/j.1440-1843.2010.01722.x PMC7192224

[7] Russell E, Ison MG. Parainfluenza virus in the hospitalized adult. Clin Infect Dis. 2017;65:1570–6. 28591775 10.1093/cid/cix528

[8] El Feghaly RE, et al. Local production of inflammatory mediators during childhood parainfluenza virus infection. Pediatr Infect Dis J. 2010;29:26-31 . 10.1097/INF.0b013e3181d5da2a PMC3417758 20182399

[9] Chemaly RF, et al. DAS181 treatment of severe lower respiratory tract parainfluenza virus infection in immunocompromised patients: a phase 2 randomized, placebo-controlled study. 2021. p. 73. 10.1093/cid/ciab113 PMC8326557 33569576

[10] Watanabe M, et al. Effect of hemagglutinin-neuraminidase inhibitors BCX 2798 and BCX 2855 on growth and pathogenicity of sendai/human parainfluenza type 3 chimera virus in mice. Antimicrob Agents Chemother. 2009;53:3942. 19564364 10.1128/AAC.00220-09 PMC2737897

[11] Alymova IV, et al. Efficacy of the novel parainfluenza virus haemagglutinin-neuraminidase inhibitor BCX 2798 in mice - further evaluation. Antivir Ther. 2009;14:891–8. 19918093 10.3851/IMP1420 PMC2782883

[12] Guillon P, et al. Structure-guided discovery of potent and dual-acting human parainfluenza virus haemagglutinin–neuraminidase inhibitors. Nat Commun. 2014;5:1–11. 10.1038/ncomms6268 25327774

[13] Dirr L, El-Deeb IM, Chavas LMG, Guillon P, Itzstein M. Von. The impact of the butterfly effect on human parainfluenza virus haemagglutinin-neuraminidase inhibitor design. Sci Rep. 2017;7:1–10. 28674426 10.1038/s41598-017-04656-y PMC5495814

[14] Pechous RD, et al. An ex vivo human precision-cut lung slice platform provides insight into SARS-CoV-2 pathogenesis and antiviral drug efficacy. J Virol. 2024;98:e00794-24. 10.1128/jvi.00794-24 PMC11265413 38940558

[15] Huang S, et al. PPAR-γ in macrophages limits pulmonary inflammation and promotes host recovery following respiratory viral infection. J Virol. 2019;93:e00030-e119. 30787149 10.1128/JVI.00030-19 PMC6475778

[16] Hodson A, Kasliwal M, Streetly M, MacMahon E, Raj K. A parainfluenza-3 outbreak in a SCT unit: sepsis with multi-organ failure and multiple co-pathogens are associated with increased mortality. Bone Marrow Transplant. 2011;46:1545–50. 21258418 10.1038/bmt.2010.347 PMC7091637

[17] Langedijk JPM, et al. Universal paramyxovirus vaccine design by stabilizing regions involved in structural transformation of the fusion protein. Nat Commun. 2024;15:1–16. 38821950 10.1038/s41467-024-48059-w PMC11143371

[18] Sauerhering L, et al. Cyclosporin a reveals potent antiviral effects in preclinical models of SARS-CoV-2 infection. Am J Respir Crit Care Med. 2022;205:964–8. 35167409 10.1164/rccm.202108-1830LE PMC9838622

[19] Obernolte H, et al. Transcriptomic analyses reveal anti-viral responses of epithelial cells and multiple immune cell types in HRV infected human lung tissue. Eur Respir J. 2017;50:PA4126.

[20] Tan K. Sen, et al. In vitro model of fully differentiated human nasal epithelial cells infected with rhinovirus reveals epithelium-initiated immune responses. J Infect Dis. 2018;217:906–15. 29228279 10.1093/infdis/jix640

[21] Galani IE, et al. Interferon-λ Mediates Non-redundant Front-Line Antiviral Protection against Influenza Virus Infection without Compromising Host Fitness. Immunity. 2017;46:875-890.e6. 28514692 10.1016/j.immuni.2017.04.025

[22] Davidson S, et al. IFN λ is a potent anti-influenza therapeutic without the inflammatory side effects of IFN α treatment. EMBO Mol Med. 2016;8:1099–112. 27520969 10.15252/emmm.201606413 PMC5009813

[23] Hall CB. Respiratory syncytial virus and parainfluenza virus. N Engl J Med. 2001;344:1917–28. 11419430 10.1056/NEJM200106213442507

[24] Jia X, et al. High expression of oleoyl-ACP hydrolase underpins life-threatening respiratory viral diseases. Cell. 2024;187:4586-4604.e20. 39137778 10.1016/j.cell.2024.07.026

[25] Nailwal H, Chan FKM. Necroptosis in anti-viral inflammation. Cell Death Differ. 2018;26:4–13. 30050058 10.1038/s41418-018-0172-x PMC6294789

[26] Vandenabeele P, Galluzzi L, Vanden Berghe T, Kroemer G. Molecular mechanisms of necroptosis: an ordered cellular explosion. Nat Rev Mol Cell Biol. 2010;11:700–14. 20823910 10.1038/nrm2970

[27] Wronski S, et al. Rhinovirus-induced human lung tissue responses mimic chronic obstructive pulmonary disease and asthma gene signatures. Am J Respir Cell Mol Biol. 2021;65:544–54. 34181859 10.1165/rcmb.2020-0337OC PMC8641849

[28] Neuhaus V, et al. Assessment of the cytotoxic and immunomodulatory effects of substances in human precision-cut lung slices. J Vis Exp. 2018;2018:57042. 10.3791/57042 PMC6101160 29806827

[29] Matrosovich M, Matrosovich T, Garten W, Klenk HD. New low-viscosity overlay medium for viral plaque assays. Virol J. 2006;3:1–7. 16945126 10.1186/1743-422X-3-63 PMC1564390

