# Supplementary figures and images for: Leveraging human precision cut lung slices for the study of human parainfluenza virus 3 infection
Source: Respir Res. 2025 Nov 14;26:315. doi: 10.1186/s12931-025-03335-1 (PMC12616994; doi:10.1186/s12931-025-03335-1)

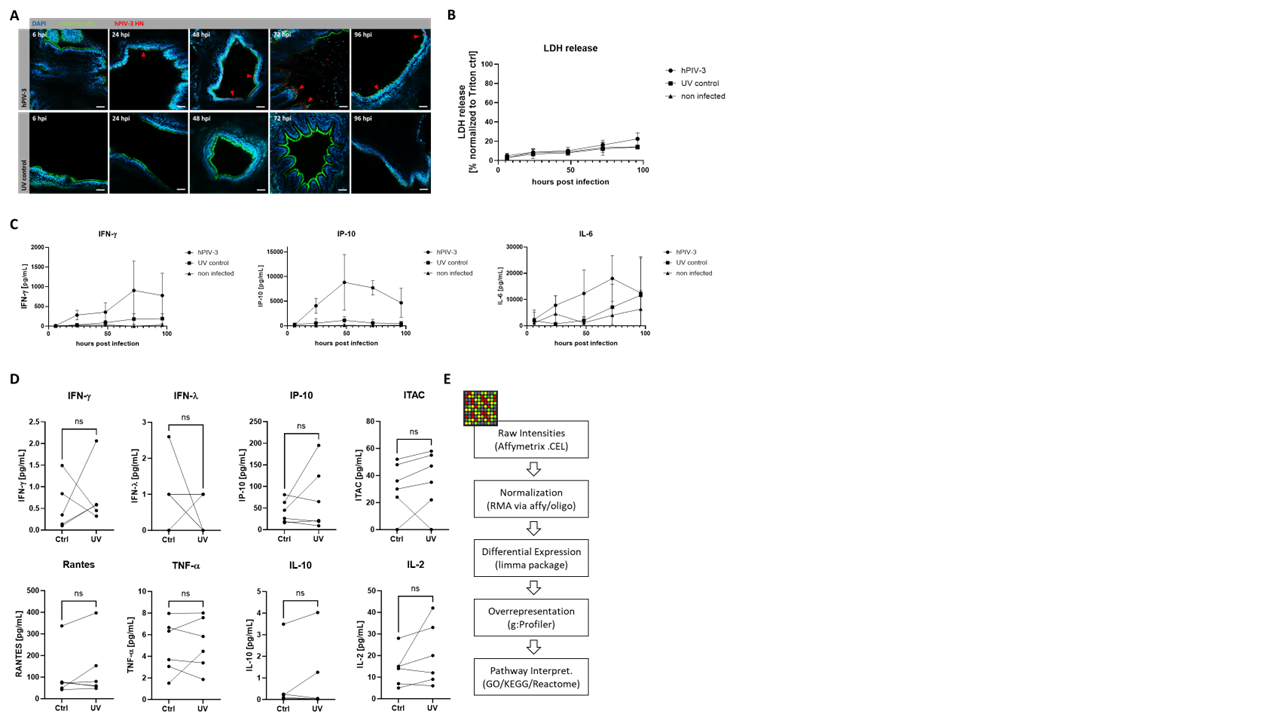

Supplement: Supplementary file 1 — Supplementary Material 1: Figure S1. A Exemplary confocal microscopy images of human PCLS infected hPIV-3 and UV-inactivated virus at 6, 24, 48, 72, and 96 hours post infection. hPIV-3-HN, ciliated cell protein Arl13b, and nucleiwere stained. Scale = 50 µm. Red arrows indicate infected patches of ciliated cells. B hPIV-3 infection only causes a cytopathic effect in human precision-cut lung slices after 96 hours post infection. LDH release from human tissue slices was measured 6, 24, 48, 72, and 96 hours post infection with medium control, UV-inactivated, or unmodified hPIV-3. An increased LDH release in infected slices compared to medium and UV control could be observed after 96 hours. C Kinetics of interferon response towards the infection.IFN-γ, IP-10, and IL-6 protein release from human tissue slices were measured 6, 24, 48, 72, and 96 hours post infection with medium control, UV-inactivated, or unmodified hPIV-3, via ELISA. Peak protein levels were observed after 72 hours post infection. b UV-inactivation renders hPIV-3 unable to induce an antiviral and pro-inflammatory response in human precision-cut lung slices. An aliquot of hPIV-3 was UV-inactivated during simultaneous cooling for 2 hours. The infection was performed according to non-inactivated virus. Cytokine secretion of selected cytokineswas measured from PCLS of 6 independent human donors. No significant increase in cytokine release was observed between UV-inactivated and non-infected control for any of the cytokines. E Exemplary ontology analysis outline. Raw data were processed and over-representation analysis for pathways and Gene Ontology Terms was performed using the package “g:profiler2”. [file 12931_2025_3335_MOESM1_ESM.tif]
